# Investigation of cell mechanics using single-beam acoustic tweezers as a versatile tool for the diagnosis and treatment of highly invasive breast cancer cell lines: an in vitro study

**DOI:** 10.1038/s41378-020-0150-6

**Published:** 2020-06-01

**Authors:** Hae Gyun Lim, Hsiao-Chuan Liu, Chi Woo Yoon, Hayong Jung, Min Gon Kim, Changhan Yoon, Hyung Ham Kim, K. Kirk Shung

**Affiliations:** 1 Department of Creative IT Engineering, Pohang University of Science and Technology, Pohang, 37673 Republic of Korea; 2 NIH Resource Center for Medical Ultrasonic Transducer Technology and Department of Biomedical Engineering, University of Southern California, Los Angeles, CA 90089 USA; 3 Department of Biomedical Engineering, Inje University, Gimhae, Gyeongnam 50834 Republic of Korea

**Keywords:** Engineering, Materials science

## Abstract

Advancements in diagnostic systems for metastatic cancer over the last few decades have played a significant role in providing patients with effective treatment by evaluating the characteristics of cancer cells. Despite the progress made in cancer prognosis, we still rely on the visual analysis of tissues or cells from histopathologists, where the subjectivity of traditional manual interpretation persists. This paper presents the development of a dual diagnosis and treatment tool using an in vitro acoustic tweezers platform with a 50 MHz ultrasonic transducer for label-free trapping and bursting of human breast cancer cells. For cancer cell detection and classification, the mechanical properties of a single cancer cell were quantified by single-beam acoustic tweezers (SBAT), a noncontact assessment tool using a focused acoustic beam. Cell-mimicking phantoms and agarose hydrogel spheres (AHSs) served to standardize the biomechanical characteristics of the cells. Based on the analytical comparison of deformability levels between the cells and the AHSs, the mechanical properties of the cells could be indirectly measured by interpolating the Young’s moduli of the AHSs. As a result, the calculated Young’s moduli, i.e., 1.527 kPa for MDA-MB-231 (highly invasive breast cancer cells), 2.650 kPa for MCF-7 (weakly invasive breast cancer cells), and 2.772 kPa for SKBR-3 (weakly invasive breast cancer cells), indicate that highly invasive cancer cells exhibited a lower Young’s moduli than weakly invasive cells, which indicates a higher deformability of highly invasive cancer cells, leading to a higher metastasis rate. Single-cell treatment may also be carried out by bursting a highly invasive cell with high-intensity, focused ultrasound.

## Cancer: An acoustic approach to diagnosis and treatment

A new acoustic technique is able to detect and kill invasive cancer cells. The technology, known as single-beam acoustic tweezers (SBAT), was developed by an international team led by Hyung Ham Kim of Korea’s Pohang University of Science and Technology. It uses an ultrasonic beam to trap cells in place and apply force to deform them. The team first calibrated SBAT with agarose gel spheres before using it to measure the deformability of breast cancer cells. The technique was able to distinguish between highly- and weakly-invasive based on their deformability, making it possible to rapidly characterize the metastatic potential of cancer cells. Finally, the researchers showed that a higher frequency beam could be used to burst highly-invasive cancer cells and that the beam could be focused well enough to target a single cell.

## Introduction

Histological image analysis is a primary clinical diagnosis method for cancer cell identification. Histopathologists and clinicians decide the malignancy level after visual screening based on the patterns and regularities of cell shapes and distributions and on the presence of molecular markers^1^. However, manual analysis and intervention are considered subjective and time-consuming and can easily generate observational variation among experts due to the complex structures of the histology images and the variability in imaging techniques and analysis protocols^2–4^. To eliminate subjective perspectives, many computer-assisted automated diagnosis systems have been developed, forming the new field of quantitative histology^5,6^. In addition, a more precise quantitative approach that directly measures the cell characteristics, including the mechanical properties, is gaining scientific importance, since it is becoming more critical to understand the functioning of a living organism at the cellular level.

The mechanical properties of biomaterials, tissues, and cells have been extensively investigated for their tissue responses and cellular functions and regulations^7,8^. The cytoskeleton, the most important cellular mechanical component, can mediate the cell response by changing its biomechanical environment, such as cell shape, cell deformation or external pressure^9^. Various blood disorders and infectious diseases are also closely linked to the morphology and biomechanical characteristics of red blood cells (RBCs)^10^. In addition, many studies have demonstrated that the mechanical stiffness level of cancer cells indicates their invasion potential, and therefore, investigating cell mechanics could play a key role in predicting and evaluating such diseases in medical and clinical fields^11^.

Currently available tools used for measuring the mechanical properties of a single cell include atomic force microscope (AFM), optical tweezers (OT), magnetic tweezers, and stretchable substrates^12^; however, there are some drawbacks to directly applying these methods to cellular-level studies. An AFM consists of a cantilever with probes that make direct contact with the surface of a cell to perform force spectroscopy measurements, which can cause the destruction of the cell membrane^13^. OT are a well-established, noncontact tool for manipulating cells and nano/microparticles with a focused laser beam. OT were used for investigating RBC elasticity^14^ and aggregation force^15,16^ by pulling a microbead attached to RBCs across the cell surface. However, because the force generated by an optical trap is limited to the order of piconewtons, it is challenging to use OT to examine the mechanical properties of the cells that require stronger forces than the maximal optical trapping force. Alternatively, magnetic tweezers are an experimental manipulation tool that use magnetic beads to measure cellular forces and the local viscoelasticity in living cells^17,18^. One of their shortcomings, however, is that the magnetic beads need to be loaded inside of a cell^17^, which would be a major limiting factor in applying the tool to in vivo measurements.

To more reliably estimate a cell’s mechanical properties, the ideal tool needs to be contactless with respect to the cell surface and should have the capability to produce stronger forces. Single-beam acoustic tweezers (SBAT) are an advanced noncontact tool that can generate a trapping force in the range from piconewtons to nanonewtons^19–21^ and a radiation pressure of several megapascals (MPa)^22,23^. For these reasons, precise cell manipulation with SBAT has been proposed for studying cellular activity in a suspension without any labeling or material attached, which would allow more practical applications for biomedicine^24,25^. Recently, SBAT with a 200 MHz ultrasonic transducer were used to measure the elastic properties of cultured breast cancer cells^26^. The cell membrane was stretched by a microbead attached to the target cell with an acoustic trap, resulting in local membrane deformation. More recently, the deformability of various cancer cells was relatively measured with acoustic trapping to differentiate degrees of cancer cell invasiveness^22^. However, the researchers could not measure the absolute value of Young’s modulus due to the lack of calibrated elasticity data for the cells. Hence, it is desirable to develop an absolute quantification method to measure the mechanical properties of cancer cells accurately and to classify cells into highly invasive or weakly invasive cells.

In this study, we demonstrated that 50 MHz SBAT can quantitate the mechanical properties of an unlabeled suspended cell. We used AHSs and cell-mimicking phantoms to calibrate the deformability of cells. A single suspended cell (detached from the culture dish but still close to the substrate) or an AHS was trapped by the SBAT, and its deformability was examined under the transducer’s various driving conditions. After a deformability experiment using SBAT, AHSs were mechanically tested using a micropipette aspiration technique (MAT) that has been used for investigating the mechanical properties of cells and cell-sized microspheres^27^. Then, the Young’s modulus of the cells was calculated and used as standardized reference data for cell deformability. With the deformability relationship between the cells and AHSs in addition to the Young’s modulus of the AHSs in 0.1~1.2% concentrations, indirect quantitative measurements of cancer cell elasticity were assessed. Furthermore, the mechanical properties of breast cancer cells with different degrees of invasiveness were quantitatively compared to classify them into highly invasive and weakly invasive cells.

In addition to sorting highly invasive cells, we developed the ability to treat or blast the cells. In this method, an excitation frequency of 70 MHz with high driving voltages was applied to damage a single cell by disrupting the cell membrane, similar to high-intensity focused ultrasound^28^ and microbubble-assisted sonoporation^29^. Since a high-frequency ultrasonic transducer can concentrate acoustic pressure into a micrometer-sized area within a single cell without microbubbles, it allows the selective killing of malignant cancer cells over their normal counterparts. These cell sorting and destroying capabilities suggest that SBAT have the potential to work as a dual diagnostic (noncontact and label-free evaluation of cell mechanics) and therapeutic (killing without micro/nanobubbles) tool for in vivo cancer treatment studies.

## Results

### Deformation of cells and AHSs with SBAT

**Fig. 1:**
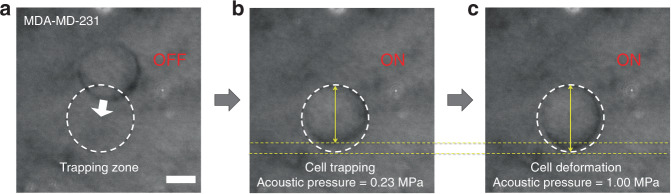
Cell trapping and deformation by the SBAT. **a** Ultrasound was off, and a MDA-MB-231 was located out of the trapping zone. **b** The cell was trapped at the trapping zone by the SBAT. **c** Cell membrane deformation under acoustic pressure. Scale bars indicate 10 μm

The SBAT have the ability to deform a trapped object, resulting in area changes along a transverse direction, as shown in Fig. 1. While the transmitted focused beam from the SBAT was applied on the cell or the AHS, its deformation was monitored, which showed that the area changes were directly proportional to the applied acoustic pressure (Fig. 1b, c and Supplementary Video 1). The pressure generated from the SBAT gradually increased from 0.0 to 1.0 MPa. The driving conditions of the SBAT were as follows: 500 cycles, pulse repetition frequency (PRF) of 1 kHz, and input voltages to the transducer of 0.00, 4.74, 9.48, 14.22, 18.96, and 23.70 peak-to-peak voltage, V_pp_ (corresponding acoustic pressures: 0.00, 0.23, 0.43, 0.63, 0.82, and 1.00 MPa).

**Fig. 2:**
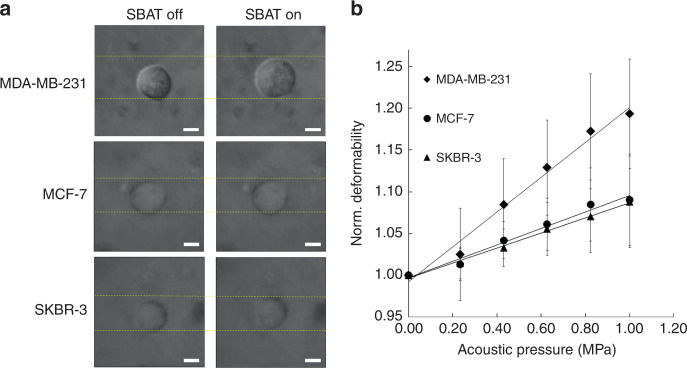
Deformability of MDA-MB-231, MCF-7, and SKBR-3 cells. **a** Bright-field images for each cell to show area changes with and without SBAT. Scale bars indicate 10 μm. **b** Normalized area changes of trapped cells at acoustic pressures of 0.00, 0.23, 0.43, 0.63, 0.82, and 1.00 MPa. The values at each cell line are the average of 20 samples

Three breast cancer cell lines, MDA-MD-231 (highly invasive), MCF-7 (weakly invasive), and SKBR-3 (weakly invasive), were compared to assess cell deformation as a function of acoustic pressure. Figure 2a presents the comparison among the shapes of the three cell types with the SBAT off and on. Figure 2b shows the area changes in each cell line as a function of the acoustic pressure (20 samples for each cell line). The area of the cells was computed using ImageJ (NIH, Bethesda, MA USA). With boundary selection, a polygon was formed around the cell, and its surface area was measured. It was found that the area of the cell increased as the acoustic pressure increased. It is clear that all three cell lines displayed the largest surface area changes at 1.0 MPa in Fig. 2a, which means that they are more deformed at higher acoustic pressures. The normalized area (the actual value divided by the maximum value among the group) of the MCF-7 cells was measured to be 1.000, 1.015, 1.041, 1.061, 1.084 and 1.090 at 0.000, 0.234, 0.431, 0.627, 0.824, and 1.000 MPa, respectively. The normalized areas of the SKBR-3 cells were 1.000 at 0.000 MPa, 1.015 at 0.234 MPa, 1.033 at 0.431 MPa, 1.055 at 0.627 MPa, 1.071 at 0.824 MPa, and 1.088 at 1.000 MPa. A very similar tendency was observed for the MCF-7 and SKBR-3 cells. In contrast, the normalized areas of the MDA-MD-231 cells were found to be considerably larger, with measurements of 1.000, 1.025, 1.085, 1.130, 1.173, and 1.194 at 0.000, 0.234, 0.431, 0.627, 0.824, and 1.000 MPa, respectively (Supplementary Table 1). The slopes of the linear regressions were calculated for each cell line by plotting the acoustic pressure versus the normalized cell deformation; as shown in Fig. 2, the cells with higher slopes exhibited more deformable properties under the SBAT.

**Fig. 3:**
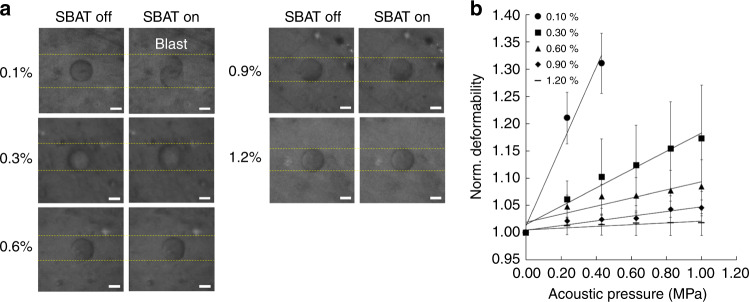
Deformability of 0.1, 0.3, 0.6, 0.9, and 1.2% agarose hydrogel spheres. **a** Bright-field images for each sphere showing area change with and without SBAT. Scale bars indicate 10 μm. **b** Normalized area changes of trapped spheres at acoustic pressures of 0.00, 0.23, 0.43, 0.63, 0.82, and 1.00 MPa. The values at each concentration are the average of 20 samples

AHSs with different agarose concentrations of 0.1 (AHSs_0.1_), 0.3 (AHSs_0.3_), 0.6 (AHSs_0.6_), 0.9 (AHSs_0.9_), and 1.2% (AHSs_1.2_) were used to assess sphere deformation under the SBAT. Since the mechanical properties of AHSs depend on their agarose concentration, these different spheres showed different deformability under SBAT. Figure 3 shows the comparison of shape changes for AHSs with five different concentrations induced by the SBAT and their deformability as a function of the acoustic pressure (*n* = 20). The normalized area of the AHSs with 0.1% concentration (i.e., AHSs_0.1_) was measured to be 1.000, 1.211, and 1.312 at acoustic pressures of 0.000, 0.234, and 0.431 MPa, respectively. Interestingly, the AHSs_0.1_ exploded when the acoustic pressure reached 0.43 MPa. Thus, we were unable to measure them further. The normalized areas of AHSs_0.3_ were 1.000, 1.061, 1.102, 1.123, 1.154, and 1.173 at 0.000, 0.234, 0.431, 0.627, 0.824, and 1.000 MPa, respectively. The normalized areas of AHSs_0.6_ were 1.000, 1.048, 1.067, 1.068, 1.077, and 1.084 at 0.000, 0.234, 0.431, 0.627, 0.824, and 1.000 MPa, respectively. The normalized areas of AHSs_0.9_ and AHSs_1.2_ at 1.000 MPa were 1.045 and 1.018, respectively (Supplementary Table 1). The slopes of the linear regressions were computed for each concentration by plotting the acoustic pressure versus the normalized sphere deformation. As shown in Fig. 3b, the slope was inversely proportional to the concentration of agarose, which means that spheres with higher agarose concentrations were less likely to be deformed. Furthermore, these results demonstrate that SBAT induced more morphological deformation in the trapped cell or sphere with stronger acoustic pressure.

### Mechanical property measurements using a MAT

**Fig. 4:**
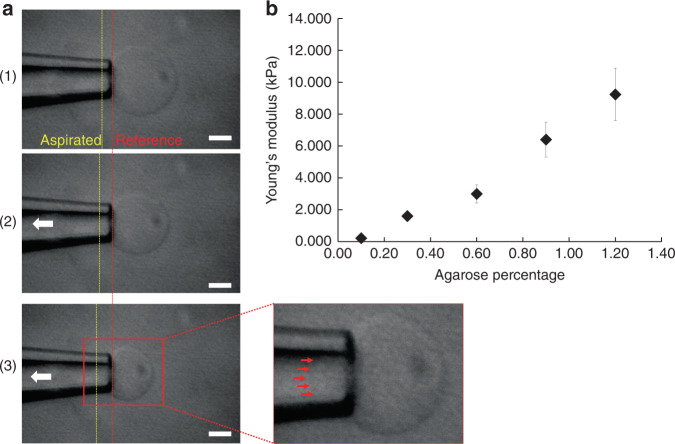
Mechanical test to investigate the Young’s modulus of 0.1, 0.3, 0.6, 0.9, and 1.2% agarose hydrogel spheres. **a** MAT was utilized to measure the mechanical properties of a 0.3% sphere. As suction pressure increased from the micropipette, the hydrogel sphere gradually deformed inside of the micropipette from (1) to (3). The red dotted line indicates how much the sphere was aspirated. Scale bars indicate 10 μm. **b** Measured young’s modulus for each sphere. Error bars indicate standard deviations. The values at each concentration are the average of 20 samples

In Fig. 4, we observed the deformation of an AHS_0.3_ using a mechanical test, the MAT. As the suction pressure increased, the AHS_0.3_ was aspirated into the micropipette, increasing the corresponding aspirated length. For given values of the geometrical, rheological and external parameters, Young’s modulus can be computed by the following expression^30^:

1 $${E} = \frac{{3R\Delta {P}}}{{2\pi D}}\phi (\eta )$$

where *E* is Young’s modulus, *R* is the inner radius of the micropipette, *ΔP* is the pressure difference, *D* is the corresponding aspirated length, and *ϕ*(*η*) is a wall function calculated using the punch model and was determined by the geometry of the micropipette. It has been reported that realistic values of η range from ~0.4 to 0.6, resulting in a mean *ϕ*(*η*) of ~2.05^30,31^. In Fig. 4 Mechanical test to investigate the Young’s modulus of 0.1, 0.3, 0.6, 0.9, 1.2% AHSs. (a) A MAT was utilized to measure the mechanical properties of a 0.3% sphere. As suction pressure increased from the micropipette, the hydrogel sphere gradually deformed inside of the micropipette from (1) to (3). The red dotted line indicates how much the sphere was aspirated. Scale bars indicate 10 μm. (b) Measured young’s modulus for each sphere. Error bars indicate standard deviations. The values at each concentration are the average of 20 samples. this study, *ϕ*(*η*) was estimated to have a mean value of 2.014 for the entire range of fabricated inner and outer micropipettes. It should be noted that it was assumed that a sphere is continuously deformable with isotropic and homogenous material properties.

**Fig. 5:**
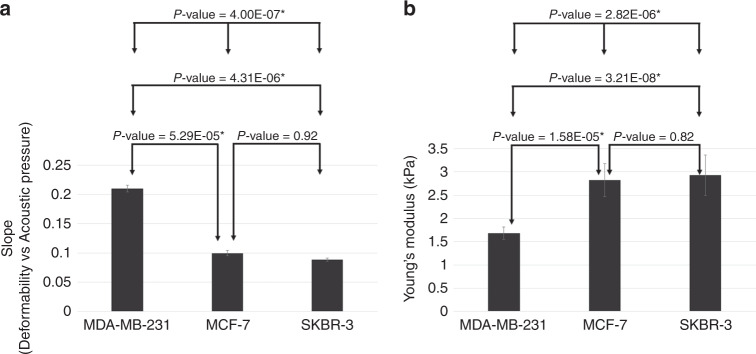
Comparison of the slope and Young’s modulus among MDA-MB-231, MCF-7, and SKBR-3 cells. ANOVA (analysis of variance) followed by the post hoc Scheffe test was used to analyze the differences in the (**a**) slopes (deformability vs acoustic pressure) and (**b**) Young’s moduli among (1) MDA-MB-231 and MCF-7, (2) MCF-7 and SKBR-3, (3) MDA-MB-231 and SKBR-3, and (4) all three cell lines. *Indicates significant differences between groups (*P*-value < 0.05)

The Young’s moduli of AHSs_0.1_, AHSs_0.3_, AHSs_0.6_, AHSs_0.9_, and AHSs_1.2_ were 0.214 ± 0.082 kPa, 1.603 ± 0.242 kPa, 2.995 ± 0.573 kPa, 6.401 ± 1.089 kPa, and 9.235 ± 1.634 kPa, respectively (Fig. 4 and Supplementary Table 1). Young’s modulus was directly proportional to the amount of agarose in the sphere. Moreover, these results were in agreement with those previously measured^32^. With the findings from Young’s modulus for each AHS, we quantified the elastic properties of the three breast cell lines. By interpolating the Young’s moduli of the AHSs into the slopes for the cells (Fig. 2) (MDA-MB-231 = 0.209, MCF-7 = 0.099, and SKBR-3 = 0.090) and for the spheres (Fig. 3) (0.1% = 0.728, 0.3% = 0.168, 0.6% = 0.075, 0.9% = 0.043, and 1.2% = 0.016), the Young’s moduli of the three breast cancer cell lines could be indirectly estimated to be 1.527 ± 0.310 kPa for MDA-MB-231, 2.650 ± 0.680 kPa for MCF-7, and 2.772 ± 0.782 kPa for SKBR-3. The slopes and Young’s moduli of the cells were compared by one-way ANOVA (analysis of variance) followed by the post hoc Scheffe test using SPSS (Statistical Package for Social Science) version 26 (Fig. 5). There was no significant difference between the values for the MCF-7 and SKBR-3 cells.

### Cell viability test

A cell viability test was performed for the MDA-MB-231, MCF-7, and SKBR-3 cells using calcein-AM (Thermo Fisher Scientific, Indianapolis, IN). Fig. S1a shows images of cell viability: positive control (treated with 0.1% bleach), control (before trapping), and negative control (after trapping). The normalized mean fluorescence representing the live-cell dye is shown in Fig. S1b, with 20 samples for each cell line. The normalized mean viabilities after trapping for the MDA-MB-231, MCF-7, and SKBR-3 cells were 1.020 ± 0.040, 1.019 ± 0.044, and 1.036 ± 0.042, respectively. There was no significant difference between the control group and the trapping group, as the *p*-values of all three cell groups were greater than 0.05 (*p* = 0.447, 0.600, and 0.335 for MDA-MB-231, MCF-7, and SKBR-3, respectively. Consequently, the trapping did not induce any significant effect on the condition of the cells under the indicated driving conditions.

### Cancer cell disruption with highly focused ultrasound

**Fig. 6:**
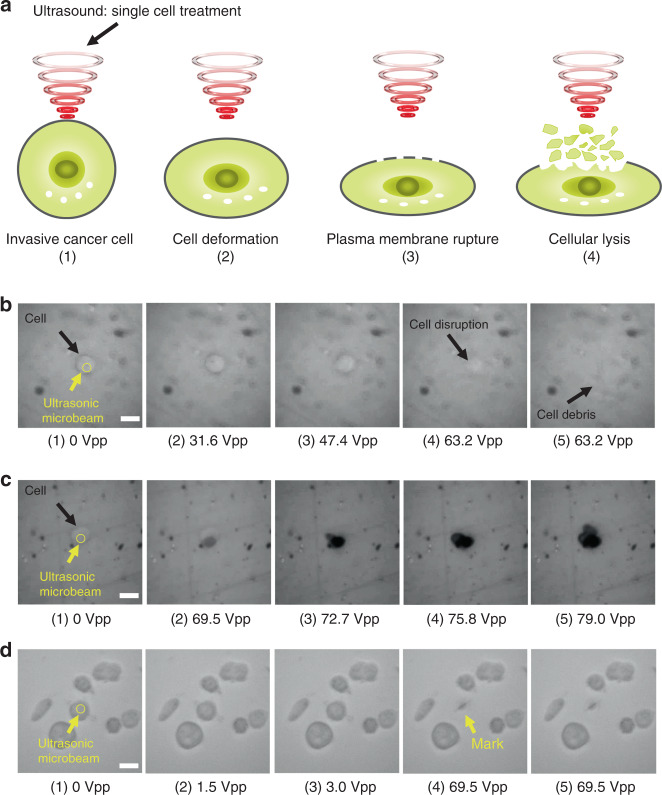
The capability of the ultrasound killer. **a** Schematic diagram of single-cell treatment using ultrasound. As the input voltage increased, the cell shows deformation, membrane rupture, and cellular lysis step-by-step. **b** The input voltage was gradually increased until cell disruption. **c** The input voltage was rapidly increased from 0 to 69.5 Vpp. A circular black mark, indicating damage to the Petri dish, was generated starting at a voltage of 69.5 Vpp. **d** A single cell was treated within a region of high cell density. Scale bars indicate 20 μm

Ultrasonic cancer cell treatment was divided into four states as follows: (1) stable state, (2) cell deformation, (3) plasma membrane rupture, and (4) cellular lysis, as shown in Fig. 6a. After measuring the Young’s modulus of the MDA-MB-213 cells using SBAT and validating the highly invasive cell itself, ultrasonic treatment of a single cancer cell was performed with high acoustic pressures. The theoretical lateral beam width (LBW) is a function of the center frequency (*f*), near field distance (*N*), element diameter (*D*), and sound velocity (*c*), as shown by Eq. 2^33^:

2 $${\mathrm{LBW}} = \frac{{Nc}}{{Df}}$$

The applied driving frequency was 70 MHz to reduce the diameter of the focused region to one corresponding to subcellular sizes (~17 μm) so that only the targeted cancer cell would be treated over their normal counterparts. Figure 6b shows the cancer cell treatment with an input voltage of 0 ~ 63.2 V_pp_, a duty factor of 1%, and a PRF of 1 kHz. The yellow circle represents the ultrasonic microbeam, and the input voltage was gradually increased, as shown in Fig. 6b1–5. The cell membrane was disrupted starting at an input voltage of 63.2 V_pp_. Figure 6c, d reveals how the single-beam ultrasonic treatment can affect the cell with higher acoustic pressure. When the input voltage reached 69.5 V_pp_, a subcellular black circular mark was generated on the Petri dish through the cell, which damaged the cell as well as the surface of the Petri dish. The size of the mark increased as the input voltage increased. In addition, Fig. 6d demonstrates the single-cell specificity of the technique by showing a single, killed target cell surrounded by multiple cells.

## Discussion

The current study demonstrated the quantification of the mechanical properties of a single cell using SBAT to deform a trapped particle or cell^22^. In this study, the absolute value of the mechanical properties, that is, the Young’s modulus of a single cell, was obtained by developing cell-mimicking phantoms with known mechanical properties. A cell-sized AHS was constructed to function as a surrogate cell and explore the feasibility of a relation between cell and AHS deformation with the SBAT. The mechanical properties of AHSs were measured by a MAT, which allowed for indirect measurements of cell elasticity. One of the main advantages of the SBAT is the generation of acoustic pressure in broad ranges from kilo to megapascals. By controlling the driving conditions of the SBAT, cells and spheres could be clearly deformed by an acoustic trap at the given acoustic pressures. As a result, the absolute Young’s modulus values illustrated the metastatic potential of cancer cells for precise and rapid characterization. In recent years, cytophone^34^, photothermal, and photodynamic therapy^35^ have been developed as dual-mode tools with many advantages, including real-time diagnosis, simple operation, and low costs. In the current feasibility study, we extended the application of acoustic tweezers technology for both diagnosis and treatment. The detecting and killing of highly invasive cancers in vitro using the SBAT were successfully demonstrated in this paper. Other potential applications of the SBAT include the detection of sickle cells or RBC aggregation in the bloodstream to effectively prevent sickle cell crisis and stroke, respectively. Many studies have reported that SBAT can trap and manipulate particles inside a blood vessel-mimicking tube^36,37^. To fully demonstrate that SBAT could function as a stand-alone diagnosis and treatment tool in preventing or inhibiting metastasis in vivo, further developments in rapid diagnostics and imaging modalities are needed to supplement current drawbacks.

Biophysical methods to predict and estimate cell stiffness are widely used for understanding cellular processes linked with diseases. Additionally, a comparative analysis of cancer cell mechanics is worth applying to disease prognosis. In this study, we investigated three breast cancer cell lines with different degrees of metastatic potential: MDA-MB-231, MCF-7, and SKBR-3. Previously, direct measurements of breast cancer cell stiffness, obtained with tools including AFMs and OT, have been made. In detail, Calzado-Martin et al. compared the Young’s modulus of MDA-MB-231 cells (25 ± 13 kPa) with that of MCF-7 cells (28 ± 12 kPa)^38^. In addition, Nikkhah et al. found the Young’s moduli of MDA-MB-231 and MCF-10A (nonmalignant) cells to be 0.4 ± 0.2 kPa and 0.7 ± 0.5 kPa, respectively^39^. Using an AFM, Wang et al. estimated the Young’s modulus of MDA-MB-231 cells as 1.0 kPa, MCF-7 cells as 2.8 kPa, and SKBR-3 cells as 1.9 kPa^40^. In general, the Young’s moduli of these cells obtained by OT was a few orders of magnitude lower than that obtained by an AFM. This is because parameters including probe stiffness, loading rate, and indentation force affect the measurement^41^. MDA-MB-231, MCF-7, and SKBR-3 cells typically exhibit different mechanical properties^39–41^, and among them, MDA-MB-231 is the most aggressive cancer cell line and has high metastatic potential. On the other hand, MCF-7 and SKBR-3 are less malignant cancer cell lines with low metastatic potential. We measured the mechanical properties of individual cells from each cell line using the SBAT, which proved that the average stiffness of MDA-MB-231 cells was much lower than that of the cells from the other two lines, which is congruent with measurements obtained by other methods^38–40^. Note that the standard error in the measurement of deformability was quite high because the cell deformability depends on the variable shape and diameter of a single cell, which are far from being perfectly constant even in the same type of cell. During the deformation process, the error might lead to an underestimation of the deformability on a relatively smaller cell, particularly because less stress is applied directly to the cell. This means that the smaller the cell, the less the acoustic force exerted since the focused acoustic beam has a fixed beam width, and depending on the size of the cell, the delivered energy is not perfectly proportional. It should also be noted that the measured cell was suspended, meaning that it was slightly touching or floating right above the dish; thus, the reflected energy from the Petri dish might also cause variations. This error can be reduced by placing the cell on an agar plate filled with a gel, a weakly scattering medium. Another important aspect is that the large deviation in the measured parameters, i.e., the large error bars, is not quite different from that of other single-cell measurement techniques, including OT, AFM, and the MAT^38–42^.

As mentioned above, OT has been popularly used for mechanical characterization because they do not require any mechanical contact force with high resolution. The OT and the optical stretcher have been used as dual identical laser beams to trap two beads attached to opposite sides of a cell and then to stretch the cell by manipulating the beams. However, the few Watts of optical laser power and the optical trapping force of up to a few piconewtons might not be enough to directly deform a trapped cell. In contrast, since the SBAT can generate a stronger trapping force of up to a few hundred nanonewtons, it can push and squeeze the cell to deform it along the transverse axis. By controlling the driving conditions of the SBAT, the acoustic pressure could be adjusted depending on the stiffness of the sample. In the present study, an acoustic pressure of less than 1.0 MPa was sufficient to deform breast cancer cells and cell-mimicking spheres to distinguish their mechanical properties. As proven in the cell viability test, acoustic pressures up to 1.00 MPa did not induce a significant effect on the cell condition.

## Conclusion

We ascertained that SBAT could serve as a theragnostic platform by integrating both diagnostic and therapeutic capabilities using the same ultrasound sources to detect and kill cancer cells. The SBAT allowed the quantification of the mechanical properties of a single cell with a noncontact and nonlabeling method. The trapping force generated from the ultrasonic transducer was able to capture and deform a cell or a sphere. AHSs functioned as surrogate cells and provided standardized reference data of cell elasticity. The elastic properties of the AHSs were mechanically measured using the MAT. Based on the measurements of the deformability of the cells and AHSs in addition to the Young’s moduli of the AHSs, the absolute mechanical properties of the cell were indirectly acquired. Measurements of cancer cell elasticity, obtained to estimate its metastatic potential, may be critical for treating the disease, and the drug treatment could be highly correlated with the mechanical properties of the cancer cell to control its metastatic potential. For future work, comparative analysis of the mechanical properties between treated and untreated cell lines obtained with SBAT will be dedicated to providing a better understanding of the efficiency of drugs in combatting the invasion potential of these cells.

## Materials and methods

### Fabrication of the transducer

A high-frequency lithium niobate (LiNbO_3_) press-focused transducer was fabricated following the procedure we used previously^20^. Since the 36°-rotated Y-cut LiNbO_3_ single crystal offers a high electromechanical coupling coefficient (k_t_ ~49%) and a low dielectric permittivity (ε^s^ ~ 39), the transducer can be designed to form a large aperture size in the material and offer a high sensitivity. The detailed procedure of this fabrication is as follows:

1. Software based on the Krimholtz, Leedom, and Matthaei model (PiezoCAD, Sonic Concepts, Bothell, WA) was used to design an optimal aperture size and thickness of LiNbO_3_, as well as matching and backing layers. LiNbO_3_ was manually lapped down to 61 μm. Chrome and gold electrodes (Cr/Au, Nano-Master, TX, USA) were sputtered with a thickness of 1500 Å on the front side of the material.
2. The first matching layer, a mixture of 2–3 μm silver particles (silver; Aldrich Chemical Co., MO, USA) and Insulcast 501 epoxy (Insulcast 501, American Safety Technologies, PA, USA), was attached to the front side of the LiNbO_3_ layer and lapped down to 9 μm. Chrome and gold electrodes were sputtered on the backside of the LiNbO_3_ layer. Conductive epoxy (E-solder 3022, Von Roll Isola, USA) was attached to the backside of the material at a thickness of 1 mm.
3. The acoustic stack (LiNbO_3_ layer, matching layer, and backing layer) was turned down to a diameter of 5 mm and then concentrically placed in brass housing. The gap between the acoustic stack and the brass was filled with an insulating epoxy (Epo-tek 201, Epoxy Technologies, Billerica, MA) to prevent electrical shorts.
4. Mechanical press focusing on the surface of the LiNbO_3_ layer was performed using a heated ball bearing ball to obtain an f-number of 0.8 (focal distance of 4 mm and aperture diameter of 5 mm). The press-focused surface of the LiNbO_3_ layer was sputtered again with chrome and gold electrodes with a thickness of 1500 Å.
5. A single-lead wire was connected to the backing layer, and then an SMA electrical connector was built into the brass housing, connecting to the single-lead wire. A second matching layer, a parylene film (10.5 μm), coated the outside of the transducer using a PDS 2010 Labcoater (SCS, Indianapolis, IN, USA).

### Transducer performance

**Fig. 7:**
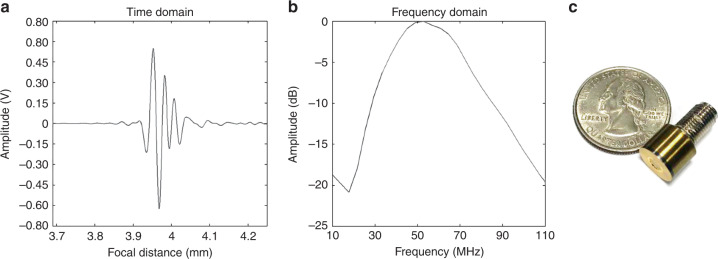
Fabrication of a highly focused 50 MHz transducer. **a** Receive-echo response. **b** Frequency spectrum. **c** Photograph of the 50 MHz transducer

To assess the performance of the fabricated transducer, a JSR (Pittsford, NY, USA) model DPR 500 pulser/receiver was connected to the transducer and produced electrical impulses at a 500 Hz repetition rate and a damping ratio of 50. The pulse-echo response and the frequency spectrum were measured as shown in Fig. 7. The center frequency was 50 MHz, and the -6 dB fractional bandwidth was 80%. The two-dimensional lateral and axial intensities of the spatial peak temporal average (I_SPTA_) was measured using a needle hydrophone (Precision Acoustics, UK), as shown in Fig. S2. The driving conditions were as follows: frequency of 50 MHz, input peak to peak voltage of 25 V, cycle number of 10, and PRF of 1 kHz. The -3 dB LBW was measured to be 32 μm. The beam profile and I_SPTA_ were observed to be symmetric at the focal point in the lateral axis.

### Cell preparation

Human breast cancer cell lines MDA-MB-231, MCF-7, and SKBR-3 were purchased from ATCC (Manassas, VA, USA) and maintained in modified complete medium (RPMI, 10% fetal bovine serum, 10 mM HEPES, 2 mM L-glutamine, 1 mM sodium-pyruvate, 0.05 mM 2-mercaptoethanol, 11 mM D-glucose). Phosphate buffer solution (PBS) was purchased from Invitrogen (Grand Island, NY) for washing the cells during the experiment. A trypsin-ethylenediaminetetraacetic acid (trypsin-EDTA) solution obtained from Invitrogen (Grand Island, NY) was used to detach the cells from the bottom of the Petri dish for suspension. Trypsin-EDTA was added, and the culture dish was incubated at 37 °C for <5 min. The trypsin incubation time is crucial in maintaining the integrity of the microtubules within the cell, but a 10-min incubation will ensure that the microtubules inside the cells remain intact instead of disintegrating^43,44^. Following the detaching process, an equivalent volume of media was added to neutralize the trypsin. This step was followed by washing the cells with PBS. As an additional precaution, in our experiments, cells with blebs (bulges of the plasma membrane) were not selected for measurements. Through this cell separation and neutralization process, we were able to separate a single cell from adherent cells and keep it suspended, i.e., the whole cell slightly touching or floating on the Petri dish without morphological distortion. The cell viability test conducted following the tweezer experiment confirmed that there was no significant adverse effect on the cell condition during the experiment; therefore, we believe that trypsin-EDTA did not affect the cell viability.

### Principles of SBAT

The principles underlying SBAT have been reported in terms of manipulation purposes in both the Mie and Rayleigh regimes. In the Mie regime, when a Gaussian acoustic beam is made incident on the object (the diameter of which is much larger than the wavelength of sound), an approaching ray can be refracted throughout the object. Due to the intensity difference between exciting rays, the gradient force in the direction of the acoustic beam axis pulls the object toward the center of the beam^25,45,46^. In contrast, particles with diameters smaller than the wavelength can also be trapped by SBAT in the Rayleigh regime^26,47^. Our proposed configuration makes use of approximate methods such as Rayleigh scattering suitable for small particles (radius < 0.4λ, λ: wavelength). As the ultrasound beam covers the whole cell area, nonhomogeneous deformation under stress was not predicted.

### Cell and sphere deformation by SBAT

**Fig. 8:**
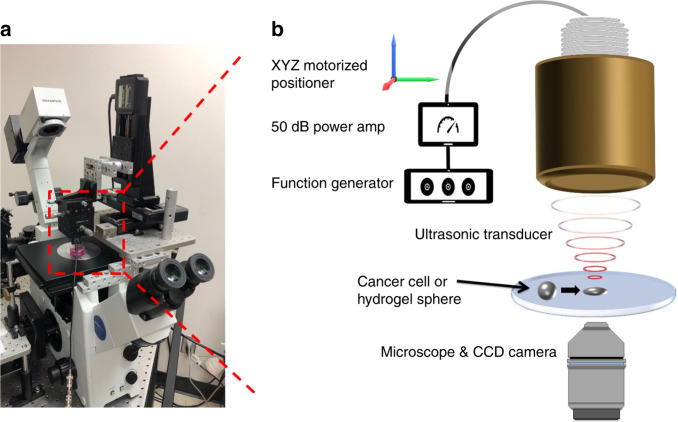
Schematic diagram of the experimental system. **a** Photograph of the experimental system. **b** The SBAT was driven at 50 MHz by sinusoidal bursts from a function generator amplified with a 50 dB amplifier. A single cell or a single sphere could be deformed by the SBAT

AHSs in 0.1, 0.3, 0.6, 0.9, and 1.2% concentrations were purchased from Particle-works (Royston, United Kingdom). The average diameter of the cells and AHSs is ~20 μm, so the amount of pressure exerted on a cell and an AHS is also similar. To generate the mechanical ultrasonic wave on the surface of a cell or a sphere, an acoustic tweezers system is required, as shown in Fig. 8. The movement of the transducer was controlled by a three-axis motorized stage (SGSP 20, Sigma KOKI Co., Japan), and the focal point on the mylar film was aligned using a pulser-receiver (5910PR; Olympus, Center Valley PA, USA). After alignment of a focal spot, a 50 MHz sinusoidal burst signal, generated by a function generator (Stanford Research Systems, Sunnyvale, CA, USA) and amplified by a 50 dB power amplifier (525LA, ENI, Rochester, USA), was driven on the transducer to grab and deform the suspended target. The duty cycle and PRF were set to 500 cycles and 1 ms, respectively. The input peak-to-peak voltage was set to 0.00, 4.74, 9.48, 14.22, 18.96, or 23.70 V. The acoustic trapping and cell deformation were observed by an inverted microscope (Olympus IX-71, Center Valley, PA, USA) and recorded via a CMOS camera (ORCA-Flash2.8, Hamamatsu, Japan).

### Mechanical test of hydrogel spheres

The AHSs were mechanically tested using the MAT. Micropipette aspiration is a well-known technique for mechanically measuring small samples, such as cells or cell-sized particles. However, this technique requires the micropipette to be in direct contact with the sample, which might cause it physical damage. For this reason, we preferred not to take direct measurement of cells using the MAT. A glass capillary containing a filament (GD-1, Narishige, NY) was heated in the middle by a vertical micropipette puller (PC-10, Narishige, NY). When the glass started to melt, the two halves of the glass were pulled apart to form micropipettes with the desired inner diameter. This micropipette was connected to a pressure controller (ez-gSEAL 100B, Neo biosystem, CA) to accurately generate suction pressure in the range from 0.0 to 33.3 kPa. The suction pressure was controlled by ez-gSEAL control software (NBSC Controller, Neo biosystem, CA). As suction pressure from the micropipette developed and then increased, a hydrogel sphere attached to the tip of the micropipette and started to deform. The deformability was varied depending on the mechanical properties of the sphere. Sphere deformation was observed using the inverted microscope and recorded via a CMOS camera.

### Cell viability test

Calcein-AM (Invitrogen, Carlsbad, CA, USA), a membrane-permeable live-cell labeling dye, was used to examine cell viability following SBAT manipulation. The cell viability was analyzed based on the cell’s fluorescence level. The cells were incubated in PBS containing 1 μM calcein-AM at room temperature for 30 min. The fluorescence level imaging of a targeted single-cell (Ex: 480 nm, Em: 530 nm) was measured before and after SBAT manipulation at 1.0 MPa. The mean and standard deviation of the fluorescence level from samples was calculated, and a two-tailed *t*-test with a level of significance of 1% was carried out.

### Single-cell treatment

The excitation frequency determines the wavelength of ultrasound generated from the transducer. To make the ultrasonic beam width smaller than the size of a single cell, the high-frequency transducer was excited with a driving frequency of 70 MHz. The duty factor and PRF were fixed at 1% and 1 kHz, respectively. Only the input voltages were varied in the single-cell treatment experiment. Sinusoidal burst signals of 70 MHz generated by a function generator and amplified by a 50 dB power amplifier with input voltages from 0 to 63.2, 79.0, and 69.5 V_pp_ were applied as shown in Fig. 6b–d, respectively (see Supplementary Video 2 for details).

## Supplementary information

Supplementary Information

Supplementary Video 1

Supplementary Video 2

Supplementary Figure

## References

[1] Nam KW (2015). Analysis of the clinical and histopathological patterns of 100 consecutive cases of primary cutaneous melanoma and correlation with staging. Arch. Plast. Surg..

[2] Wang F (2011). A data model and database for high-resolution pathology analytical image informatics. J. Pathol. Inf..

[3] Nelissen BG, van Herwaarden JA, Moll FL, van Diest PJ, Pasterkamp G (2014). SlideToolkit: an assistive toolset for the histological quantification of whole slide images. PLoS ONE.

[4] Zerbe N, Hufnagl P, Schlüns K (2011). Distributed computing in image analysis using open source frameworks and application to image sharpness assessment of histological whole slide images. Diagn. Pathol..

[5] Tan T (2013). Computer-aided detection of cancer in automated 3-D breast ultrasound. IEEE Trans. Med. Imaging.

[6] Win KY (2018). Computer aided diagnosis system for detection of cancer cells on cytological pleural effusion images. Biomed. Res. Int..

[7] Titushkin I, Cho M (2009). Regulation of cell cytoskeleton and membrane mechanics by electric field: role of linker proteins. Biophys. J..

[8] Deguchi S, Sato M (2009). Biomechanical properties of actin stress fibers of non-motile cells. Biorheology.

[9] Janmey PA, McCulloch CA (2007). Cell mechanics: integrating cell responses to mechanical stimuli. Annu. Rev. Biomed. Eng..

[10] Diez-Silva M, Dao M, Han J, Lim CT, Suresh S (2010). Shape and biomechanical characteristics of human red blood cells in health and disease. MRS Bull..

[11] Swaminathan V (2011). Mechanical stiffness grades metastatic potential in patient tumor cells and in cancer cell lines. Cancer Res..

[12] Bao G, Suresh S (2003). Cell and molecular mechanics of biological materials. Nat. Mater..

[13] Rico F (2005). Probing mechanical properties of living cells by atomic force microscopy with blunted pyramidal cantilever tips. Phys. Rev. E Stat. Nonlin. Soft Matter Phys..

[14] Hénon S, Lenormand G, Richert A, Gallet F (1999). A new determination of the shear modulus of the human erythrocyte membrane using optical tweezers. Biophys. J..

[15] Lee K (2016). Optical tweezers study of red blood cell aggregation and disaggregation in plasma and protein solutions. J. Biomed. Opt..

[16] Lee K, Danilina AV, Kinnunen M, Priezzhev AV, Meglinski I (2016). Probing the red blood cells aggregating force with optical tweezers. IEEE J. Sel. Top. Quantum Electron..

[17] Bausch AR, Möller W, Sackmann E (1999). Measurement of local viscoelasticity and forces in living cells by magnetic tweezers. Biophys. J..

[18] Walter N, Selhuber C, Kessler H, Spatz JP (2006). Cellular unbinding forces of initial adhesion processes on nanopatterned surfaces probed with magnetic tweezers. Nano Lett..

[19] Li Y, Lee C, Ho Lam K, Kirk Shung K (2013). A simple method for evaluating the trapping performance of acoustic tweezers. Appl. Phys. Lett..

[20] Lim HG (2016). Calibration of trapping force on cell-size objects from ultra-high frequency single beam acoustic tweezer. IEEE Trans. Ultrason. Ferroelectr. Freq. Control.

[21] Lim HG, Shung KK (2017). Quantification of inter-erythrocyte forces with ultra-high frequency (410 MHz) single beam acoustic tweezer. Ann. Biomed. Eng..

[22] Hwang JY (2016). Cell deformation by single-beam acoustic trapping: a promising tool for measurements of cell mechanics. Sci. Rep..

[23] Lee J, Lee C, Shung KK (2010). Calibration of sound forces in acoustic traps. IEEE Trans. Ultrason. Ferroelectr. Freq. Control.

[24] Hwang JY (2015). Acoustic tweezers for studying intracellular calcium signaling in SKBR-3 human breast cancer cells. Ultrasonics.

[25] Lam KH (2016). Multifunctional single beam acoustic tweezer for non-invasive cell/organism manipulation and tissue imaging. Sci. Rep..

[26] Hwang JY (2014). Cell membrane deformation induced by a fibronectin-coated polystyrene microbead in a 200-MHz acoustic trap. IEEE Trans. Ultrason. Ferroelectr. Freq. Control.

[27] Lee LM, Liu AP (2014). The application of micropipette aspiration in molecular mechanics of single cells. J. Nanotechnol. Eng. Med..

[28] Guan L, Xu G (2016). Damage effect of high-intensity focused ultrasound on breast cancer tissues and their vascularities. World J. Surg. Oncol..

[29] Fan Z, Kumon RE, Deng CX (2014). Mechanisms of microbubble-facilitated sonoporation for drug and gene delivery. Ther. Deliv..

[30] Theret DP, Levesque MJ, Sato M, Nerem RM, Wheeler LT (1988). The application of a homogeneous half-space model in the analysis of endothelial cell micropipette measurements. J. Biomech. Eng..

[31] Boudou T (2006). An extended modeling of the micropipette aspiration experiment for the characterization of the Young’s modulus and Poisson’s ratio of adherent thin biological samples: numerical and experimental studies. J. Biomech..

[32] Walker JM (2011). Nondestructive evaluation of hydrogel mechanical properties using ultrasound. Ann. Biomed. Eng..

[33] Lam KH (2013). Ultrahigh frequency lensless ultrasonic transducers for acoustic tweezers application. Biotechnol. Bioeng..

[34] Galanzha, E. I. et al. In vivo liquid biopsy using Cytophone platform for photoacoustic detection of circulating tumor cells in patients with melanoma. *Sci. Transl. Med.* **11**, 10.1126/scitranslmed.aat5857 (2019). 10.1126/scitranslmed.aat5857 PMC9235419 31189720

[35] Wang Y (2018). Functionalized Cu 3 BiS 3 nanoparticles for dual-modal imaging and targeted photothermal/photodynamic therapy. Nanoscale.

[36] Li Y, Lee C, Chen R, Zhou Q, Shung KK (2014). A feasibility study of in vivo applications of single beam acoustic tweezers. Appl. Phys. Lett..

[37] Lee J, Jeong JS, Shung KK (2013). Microfluidic acoustic trapping force and stiffness measurement using viscous drag effect. Ultrasonics.

[38] Calzado-Martín A, Encinar M, Tamayo J, Calleja M, San Paulo A (2016). Effect of actin organization on the stiffness of living breast cancer cells revealed by peak-force modulation atomic force microscopy. ACS Nano.

[39] Nikkhah M, Strobl JS, Schmelz EM, Agah M (2011). Evaluation of the influence of growth medium composition on cell elasticity. J. Biomech..

[40] Wang B, Guo P, Auguste DT (2015). Mapping the CXCR4 receptor on breast cancer cells. Biomaterials.

[41] Coceano G (2016). Investigation into local cell mechanics by atomic force microscopy mapping and optical tweezer vertical indentation. Nanotechnology.

[42] Cross SE (2008). AFM-based analysis of human metastatic cancer cells. Nanotechnology.

[43] Badley RA, Woods A, Carruthers L, Rees DA (1980). Cytoskeleton changes in fibroblast adhesion and detachment. J. Cell Sci..

[44] Yu H (2010). Mechanical behavior of human mesenchymal stem cells during adipogenic and osteogenic differentiation. Biochem. Biophys. Res. Commun..

[45] Lee J (2009). Single beam acoustic trapping. Appl. Phys. Lett..

[46] Kim MG (2017). Label-free analysis of the characteristics of a single cell trapped by acoustic tweezers. Sci. Rep..

[47] Lim HG, Kim HH, Yoon C, Shung KK (2020). A One-Sided Acoustic Trap for Cell Immobilization Using 30-MHz Array Transducer. IEEE Trans. Ultrason. Ferroelectr. Freq. Control.

