# Supplementary material for: Investigation of cell mechanics using single-beam acoustic tweezers as a versatile tool for the diagnosis and treatment of highly invasive breast cancer cell lines: an in vitro study
Source: Microsyst Nanoeng. 2020 Jun 1;6:39. doi: 10.1038/s41378-020-0150-6 (PMC8433385; doi:10.1038/s41378-020-0150-6)
Supplement: Supplementary file 1 — Supplementary Information [file 41378_2020_150_MOESM1_ESM.docx]

Investigation of cell mechanics using single-beam acoustic tweezer as a versatile tool for diagnosis and treatment of highly invasive breast cancer cell lines: an in vitro study

Hae Gyun Lim^1^, Hsiao-Chuan Liu^2^, Chi Woo Yoon^2^, Hayong Jung^2^, Min Gon Kim^2^, Changhan Yoon^3^, Hyung Ham Kim^1*^, and K. Kirk Shung^2^

^1^Department of Creative IT Engineering, Pohang University of Science and Technology, Pohang 37673, Republic of Korea

^2^NIH Resource Center for Medical Ultrasonic Transducer Technology and Department of Biomedical Engineering, University of Southern California, Los Angeles, California, 90089, USA

^3^Department of Biomedical Engineering, Inje University, Gimhae, Gyeongnam, 50834, Republic of Korea

*Correspondence should be addressed to H.H.K. (david.kim@postech.ac.kr)

Department of Creative IT Engineering, Pohang University of Science and Technology

77 Cheongam-ro, Nam-gu, C5 building #209

Pohang, Gyeongbuk, 37673, Republic of Korea

Phone: +82-54-279-8864

**Supplementary Materials**

**Supplementary Figure 1. Cell viability test of MDA-MB-231, MCF-7, and SKBR-3 cells. a** fluorescence images for MDA-MB-231, MCF-7, and SKBR-3 cells for before SBAT (negative control), after SBAT (experimental group), and 0.1% bleach test (positive control). Scale bars indicate 10 μm. **b** Normalized fluorescence intensity of cells before and after SBAT. Error bars indicate standard deviations. Sample number for each cell was 20.

**Supplementary Figure 2. Acoustic pressure field of the ultrasonic transducers measured by a needle hydrophone.** **a** One-dimensional lateral intensity of spatial peak temporal average (I_SPTA_) was measured. The -3 dB lateral beam width was measured to be 32 μm. **b** 2D acoustic pressure field was measured after a 50 MHz transducer was excited with the input parameters of V_pp_= 25 V, cycle numbers of 10, and pulse repetition frequency of 1 kHz.

**Supplementary Table 1. Normalized deformability and measured Young’s modulus of cell-mimicking spheres (0.1, 0.3, 0.6, 0.9, and 1.2%) and cancer cells (MDA-MB-231, MCF-7, and SKBR-3).**

**Supplementary Video Captions**

**Supplementary Video 1. Cell trapping and deformation after the application of the SBAT.** When the acoustic tweezer was turned on, a MDA-MB-231 was manipulated and trapped into the trapping zone. The cell boundary was deformed with input peak to peak voltage from 0 ~ 20 V, cycle numbers of 500, and pulse repetition frequency of 1 kHz. Total duration: 30 seconds.

**Supplementary Video 2. Killing of a highly invasive cancer cell by ultrasound.** A MDA-MB-231 was killed by a strong acoustic pressure. When input peak to peak voltage reached 69.5 V, a black circular mark was generated on the surface of the petri dish, and the cell was killed simultaneously. In this video, input voltage was rapidly increased from 0 to 69.5 Vpp in a one-step, then gradually increased up to 79.0 V_pp_ with cycle numbers of 500 and pulse repetition frequency of 1 kHz. Total duration: 8 seconds.

**Supplementary Figure 1.**

**
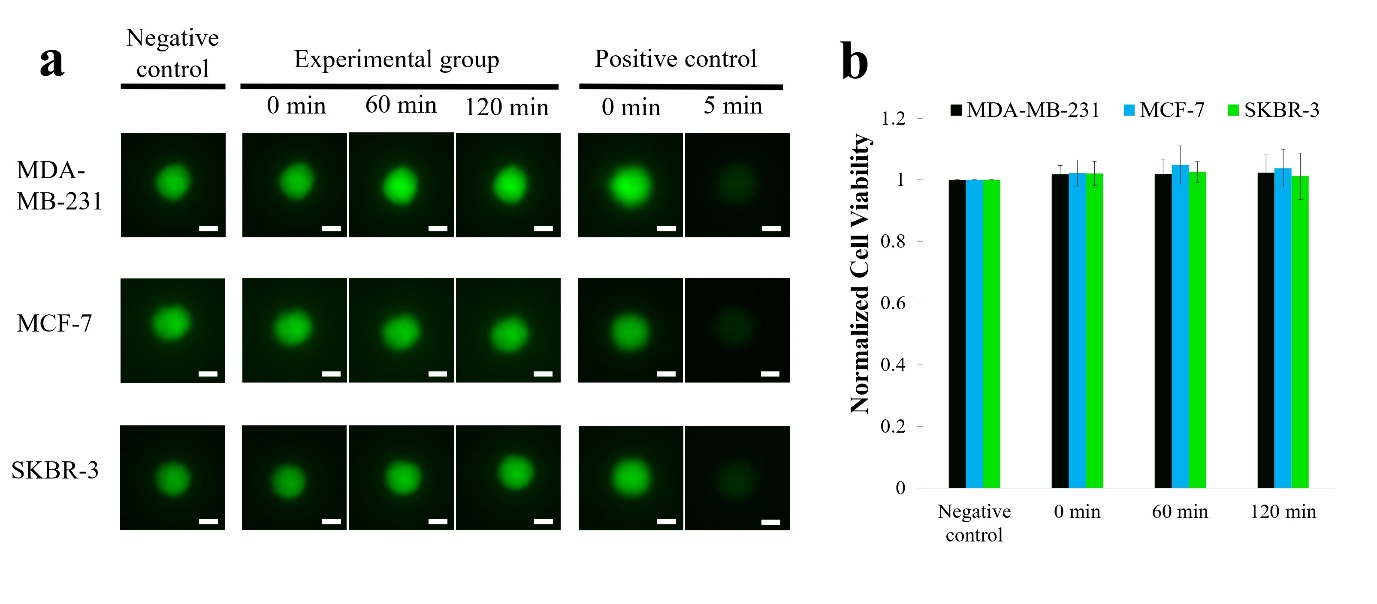
**

**Supplementary Figure 2.**

**
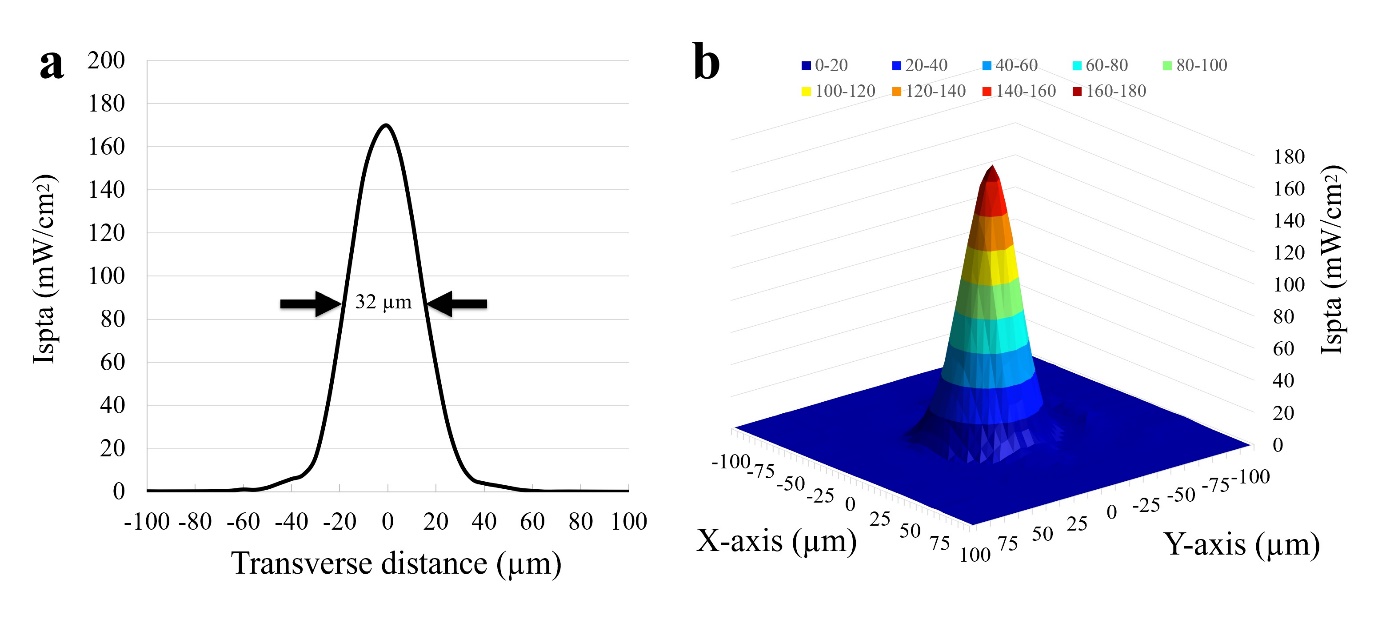
**

**Supplementary Table 1.**

|  | | Normalized deformability | | | | | **Young’s modulus** |
| --- | --- | --- | --- | --- | --- | --- | --- |
| **Acoustic pressure (Mpa)** | | **0.23** | **0.43** | **0.63** | **0.82** | **1.00** |  |
| **Cell-mimicking sphere** | **0.1%** | 1.211 ± 0.047 | 1.312 ± 0.055 | X | X | X | 0.214 ± 0.082 |
|  | **0.3%** | 1.061 ± 0.034 | 1.102 ± 0.069 | 1.123 ± 0.074 | 1.154 ± 0.086 | 1.173 ± 0.098 | 1.603 ± 0.242 |
|  | **0.6%** | 1.048 ± 0.021 | 1.067 ± 0.022 | 1.068 ± 0.033 | 1.077 ± 0.038 | 1.084 ± 0.049 | 2.995 ± 0.573 |
|  | **0.9%** | 1.021 ± 0.006 | 1.024 ± 0.008 | 1.026 ± 0.011 | 1.043 ± 0.015 | 1.045 ± 0.014 | 6.401 ± 1.089 |
|  | **1.2%** | 1.012 ± 0.017 | 1.015 ± 0.019 | 1.017 ± 0.023 | 1.018 ± 0.023 | 1.018 ± 0.023 | 9.235 ± 1.634 |
| **Cell** | **MDA-MB-231** | 1.025 ± 0.055 | 1.085 ± 0.055 | 1.130 ± 0.057 | 1.173 ± 0.069 | 1.194 ± 0.066 | 1.527 ± 0.310 |
|  | **MCF-7** | 1.015 ± 0.012 | 1.041 ± 0.026 | 1.061 ± 0.044 | 1.084 ± 0.060 | 1.090 ± 0.059 | 2.650 ± 0.680 |
|  | **SKBR-3** | 1.015 ± 0.019 | 1.033 ± 0.022 | 1.055 ± 0.031 | 1.071 ± 0.043 | 1.088 ± 0.055 | 2.772 ± 0.782 |
