# Supplementary figures and images for: Investigation of cell mechanics using single-beam acoustic tweezers as a versatile tool for the diagnosis and treatment of highly invasive breast cancer cell lines: an in vitro study
Source: Microsyst Nanoeng. 2020 Jun 1;6:39. doi: 10.1038/s41378-020-0150-6 (PMC8433385; doi:10.1038/s41378-020-0150-6)

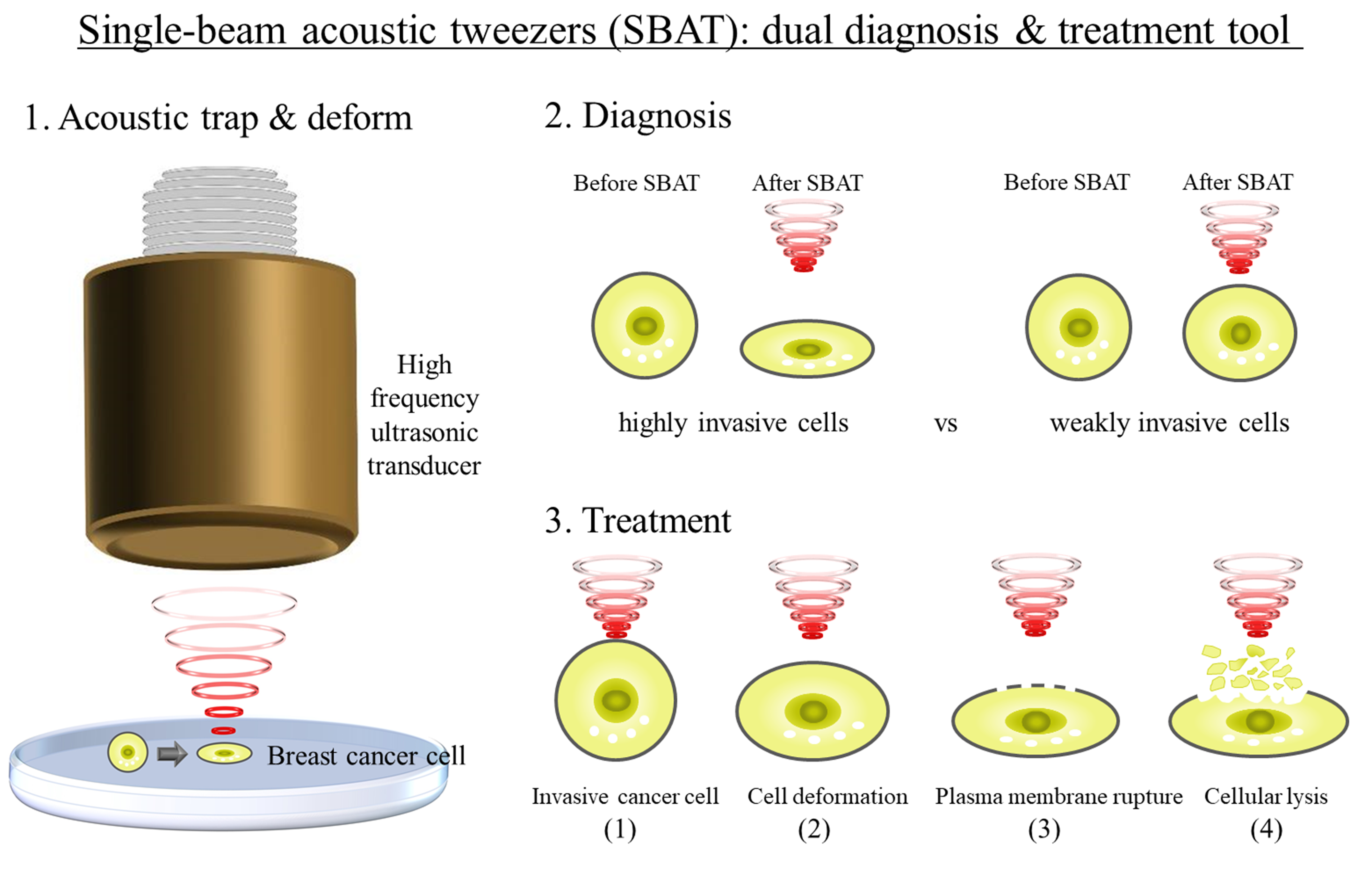

Supplement: Supplementary file 4 — Supplementary Figure [file 41378_2020_150_MOESM4_ESM.tif]
